# Origin and Formation Mechanism of Carbon Shell-Encapsulated Metal Nanoparticles for Powerful Fuel Cell Durability

**DOI:** 10.3390/nano13212862

**Published:** 2023-10-29

**Authors:** Hyeonwoo Choi, Yoonseong Choi, Jiho Min, Keonwoo Ko, Yunjin Kim, Sourabh S. Chougule, Davletbaev Khikmatulla, Namgee Jung

**Affiliations:** Graduate School of Energy Science and Technology (GEST), Chungnam National University, 99 Daehak-ro, Yuseong-gu, Daejeon 34134, Republic of Korea; snow7780@o.cnu.ac.kr (H.C.); gubongman@o.cnu.ac.kr (Y.C.); mjh9780@o.cnu.ac.kr (J.M.); kkw00000@o.cnu.ac.kr (K.K.); yunjinkim1994@o.cnu.ac.kr (Y.K.); schougule@o.cnu.ac.kr (S.S.C.); haki030899@o.cnu.ac.kr (D.K.)

**Keywords:** proton exchange membrane fuel cell, carbon shell, encapsulation, formation mechanism, durability

## Abstract

Proton exchange membrane fuel cells (PEMFCs) face technical issues of performance degradation due to catalyst dissolution and agglomeration in real-world operations. To address these challenges, intensive research has been recently conducted to introduce additional structural units on the catalyst surface. Among various concepts for surface modification, carbon shell encapsulation is known to be a promising strategy since the carbon shell can act as a protective layer for metal nanoparticles. As an interesting approach to form carbon shells on catalyst surfaces, the precursor ligand-induced formation is preferred due to its facile synthesis and tunable control over the carbon shell porosity. However, the origin of the carbon source and the carbon shell formation mechanism have not been studied in depth yet. Herein, this study aims to investigate carbon sources through the use of different precursors and the introduction of new methodologies related to the ligand exchange phenomenon. Subsequently, we provide new insights into the carbon shell formation mechanism using X-ray photoelectron spectroscopy (XPS) and X-ray diffraction (XRD). Finally, the thermal stability and electrochemical durability of carbon shells are thoroughly investigated through in situ transmission electron microscopy (in situ TEM) and accelerated durability tests.

## 1. Introduction

As the demand for devices based on electrochemical technology continues to rise, there is an increasing need for high-performance electrochemical catalysts. Among these, interest in fuel cells based on hydrogen energy is growing rapidly due to the climate crisis and the push for green energy alternatives to fossil fuels. Proton exchange membrane fuel cells (PEMFCs), which primarily use Pt-based catalysts, operate in acidic environments and face significant durability issues, such as agglomeration and dissolution, during long-term operation. Additionally, the active sites of catalysts are susceptible to changes through dissolution, detachment, phase transitions, and deposition during electrochemical processes, leading to a decrease in catalyst performance [1,2,3,4,5,6,7].

To address these challenges, extensive efforts have been made to investigate the degradation mechanisms of nanostructured catalysts under reaction conditions. The synthesis of durable catalysts, often in the form of core-shell structures, has also been proposed [8,9,10,11,12,13,14,15]. However, there has been a trade-off between achieving an active structure and a durable structure, highlighting the limitations of single-catalyst structures in terms of durability. Consequently, recent research has intensively explored the introduction of additional structural units as an approach to overcome these limitations in catalyst design. Among various proposed structures like silica shells and magnesium oxide layers, carbon shell-encapsulated nanoparticles have emerged as a promising candidate since ultrathin carbon shells can act as protective layers, preventing a decrease in catalytic activity and enhancing durability in electrochemical environments prone to oxidation [16,17,18,19,20].

Carbon shell encapsulation strategies can be classified into two types: polymer coating-based approaches and precursor ligand-induced formation. Meanwhile, the precursor ligand-induced formation offers significant advantages over polymer coating methods. It uses only traces of carbon sources (from organic ligands), allowing for well-controlled carbon shell structures with respect to shell morphology and porosity and simplifying the synthesis process. However, excessively dense shells can impede gas access to the catalyst’s active sites, potentially leading to reduced performance. Therefore, maintaining an appropriate shell thickness and porosity is crucial. Well-controlled carbon shells are the key to inhibiting structural deformation while maintaining both activity and durability. Thus, the precursor ligand-induced method holds a distinct advantage in this regard [21,22,23,24,25,26,27]. Despite this, the origin and formation mechanism of carbon shell formation through these precursor-based methods have not been clearly elucidated yet. Although numerous papers have utilized and discussed carbon shell-encapsulated nanoparticles, in-depth study on how carbon shells are truly formed has been lacking.

Herein, we aim to comprehensively investigate the origin and formation mechanism of the carbon shell to develop better structures for practical applications. Firstly, we investigate the origin of carbon shells by employing different acetylacetonate/chloride precursors. It also introduces a novel approach using surfactants and a precursor ligand exchange to uncover the source of carbon. Secondly, we try to interpret the formation mechanism of carbon shells within Pt by analyzing the process through X-ray photoelectron spectroscopy (XPS) and X-ray diffraction (XRD), shedding new light on this mechanism. Lastly, we discuss the use of gases to create well-controlled carbon shell shapes and substantiate durability improvements due to the protective layers through accelerated degradation tests (ADTs) and in situ transmission electron microscopy (TEM). This research is expected to offer valuable insights into the development of structural control strategies for electrochemical catalysts and the potential expansion of their use in thermochemical catalysts, leveraging their thermal stability [28,29].

## 2. Materials and Methods

### 2.1. Chemicals and Materials

Carbon blacks (Vulcan XC-72, Cabot, Boston, MA, USA) were purchased from Cabot Inc.; 1-Octadecene (90%), platinum acetylacetonate (Pt(acac)_2_, 97%), platinum (II) chloride (PtCl_2_, 98%), oleylamine (70%), Oleic acid (70%), Nafion ionomer (5 wt%), and 2-propanol (99.5%) were procured from Sigma-Aldrich Inc. (Sigma−Aldrich, Burlington, MA, USA). n-Hexane (95%) and ethanol (95%) were acquired from Samchun Pure Chemicals (Daejeon, Republic of Korea). A rotating disk electrode (RDE) with glassy carbon (GC, geometric area = 0.196 cm^2^) was purchased from Metrohm-Autolab (Utrecht, The Netherlands).

### 2.2. Preparation of Carbon Shell-Encapsulated Pt Nanoparticles Using Different Precursors

To synthesize the catalyst using the Pt(acac)_2_ precursor, carbon black of 0.1 g was dispersed in 1-octadecene of 140 mL by 20 min sonication. Platinum acetylacetonate (Pt(acac)_2_) of 0.053 g was dispersed in 20 mL of 1-octadecene by 20 min sonication. After the two solutions were blended and then mixed in Ar atmosphere at 120 °C for 1 h to remove impurities, such as O_2_ and moisture from the solution, the temperature of the solution was increased up to 300 °C and held for 2 h for the thermal decomposition of the Pt precursor. After finalizing the reaction, the solution was cooled down to 80 °C and then washed and filtered by copious hexane and ethanol. The as-prepared catalyst was dried in an oven at 60 °C and then annealed at 700 °C for 1 h in Ar atmosphere to form carbon shell layers on Pt nanoparticles. This catalyst is denoted as Pt_acac_/C. Using the same method, another catalyst synthesizes with platinum chloride (PtCl_2_) of 0.034 g. This catalyst was named Pt_Cl_/C. As an additional sample, the Pt(acac)_2_ precursor-based catalyst was annealed at 700 °C for 1 h in H_2_ atmosphere instead of Ar, designated as Pt_acac_/C-H_2_.

### 2.3. Preparation of Carbon Shell-Encapsulated Pt Nanoparticles Using Pt(acac)_2_ Precursor and Surfactant

For the first sample, proceeding with the same synthesis using the Pt(acac)_2_ precursor, 10 mL of oleylamine was added to the 1-octadecene solution containing the highly dispersed Pt precursor and carbon support. For the second sample, 5 mL of oleic acid and 5 mL of oleylamine were added to the solution. Each sample was then annealed at 700 °C for 1 h in Ar atmosphere. These respective samples were designated as Pt_acac-OAm_/C and Pt_acac-OAc_/C.

### 2.4. Physical Characterization

To assess the reduction extent of the synthesized Pt_acac_/C and Pt_Cl_/C catalysts, a thermogravimetric analyzer (TGA) (TGA8000, Woodbridge, ON, USA) was employed. In the TGA analysis, air flowed by increasing the temperature from room temperature to 900 °C. Additionally, the dispersion and average particle size of the prepared catalysts were determined using TEM (Tecnai G2 F30 S-Twin, FEI, Eindhoven, The Netherlands), while high-resolution TEM (HR-TEM) (Titan G2 Cube 60–300, FEI, Eindhoven, The Netherlands) was used to observe the carbon layer coated on the Pt surface. And the atomic distribution in metal nanoparticles was confirmed through scanning transmission electron microscopy (STEM) (Tecnai G2 F30 S-Twin, FEI, Eindhoven, The Netherlands)-energy dispersive X-ray spectroscopy (EDS) mapping. Furthermore, a comparison of the crystal structures of Ptacac/C and PtCl/C catalysts were carried out using XRD (SmartLab, Rigaku, Tokyo, Japan) and their crystallite sizes were analyzed using the full width at half maximum (FWHM) of the (220) plane. Changes in the electronic structure of the Pt nanoparticle surface were analyzed using XPS (K-alpha+, Thermo Scientific, East Grinstead, UK). Additionally, structural changes in the Pt/C commercial catalyst and Pt_acac_/C-H_2_ catalyst after ADTs were investigated using HR-TEM. Furthermore, real-time imaging analysis of particle aggregation and distribution changes was conducted by elevating the temperature from 25 to 900 °C using in situ TEM (HF 5000, HITACHI, Tokyo, Japan) [30,31].

### 2.5. Electrochemical Measurements

All electrochemical measurements were conducted in a conventional three-compartment electrochemical cell using an RDE, Pt wire, and Ag/AgCl electrode as the working, counter, and reference electrodes, respectively. All the potential values were represented by a reversible hydrogen electrode (RHE). Catalyst inks were prepared by mixing 5 mg of catalyst with 34.4 μL of a Nafion solution and 500 μL of 2-propanol. A drop of the catalyst ink (4 μL) was applied to the GC electrode, and when dried, we conducted the electrochemical test. Pt loading on the glassy carbon was 38.19 μg∙cm^−2^. CVs were scanned by cycling the potential between 0.05 and 1.05 V_RHE_ at 20 mV∙s^−1^ in Ar-saturated 0.1 M HClO_4_. For the ORR tests in O_2_-saturated 0.1 M HClO_4_, the potential was scanned at 5 mV∙s^−1^ between 0.05 and 1.05 V_RHE_ with a rotation speed of 1600 rpm. CO stripping tests were recorded by poisoning the Pt surface with pure CO gas followed by CO oxidation reaction. CO gas was first bubbled for catalyst poisoning into 0.1 M HClO_4_ for 15 min while holding the potential at 0.05 V_RHE_. After the electrolyte was purged with Ar gas for 20 min to completely remove the remaining CO molecules in the electrolyte, CV curves were obtained in Ar-saturated electrolyte with a scan rate of 20 mV∙s^−1^ at room temperature and in the potential range of 0.05−1.05 V_RHE_. The exposed metal surface area (EMSA) was calculated by integrating the currents in the CO oxidation peak area, presuming a monolayer CO charge of 420 μC∙cm^−2^. (The detailed calculation procedure is provided in the Supplementary Information). ADTs for the Pt/C and Pt_acac_/C-H_2_ samples were conducted by 10,000 potential cycling between 0.6 and 1.1 V_RHE_ at a scan rate of 100 mV∙s^−1^ in O_2_-saturated 0.1 M HClO_4_. After 10,000 cycles of ADTs, the CVs, CO stripping curves, and ORR curves of the catalysts were recorded again and compared with those of the catalysts before the ADTs.

## 3. Results and Discussion

### 3.1. Origin of Carbon Source

A few researchers have reported that the choice of organic ligands (e.g., acetylacetonate) has a significant impact on the catalyst when used as a metal complex in thermal decomposition synthesis [32,33,34,35]. This led us to speculate that the carbon source for the carbon shell originates from the precursor ligand. To investigate the actual origin of the carbon source, both qualitatively and quantitatively, we conducted an in-depth study in this context. 

For our experiments, we used Pt(acac)_2_ as a carbon source-containing ligand complex precursor and PtCl_2_ as a carbon source-free precursor. As Figure S1 shows, although there are various carbon source-free precursors like H_2_PtCl_6_·xH_2_O and PtCl_4_, these precursors with Pt^4+^ have a lower reduction potential of around 0.76 V, compared to the 1.18 V for Pt(acac)_2_ with Pt^2+^. This lower reduction potential makes the reduction of Pt ions relatively difficult during the synthesis at 300 °C [36,37,38]. Therefore, we chose PtCl_2_ with Pt^2+^ since it was successfully reduced to metal nanoparticles by thermal decomposition and achieved the target amount of Pt loading. The TGA clearly confirmed that the Pt_Cl_/C catalyst made with PtCl_2_ had ~20 wt% Pt loading, similar to the Pt_acac_/C catalyst made with Pt(acac)_2_, reaffirming its suitability for investigating the effect of the presence or absence of a carbon source (Figure S1d,e).

**Figure 1:**
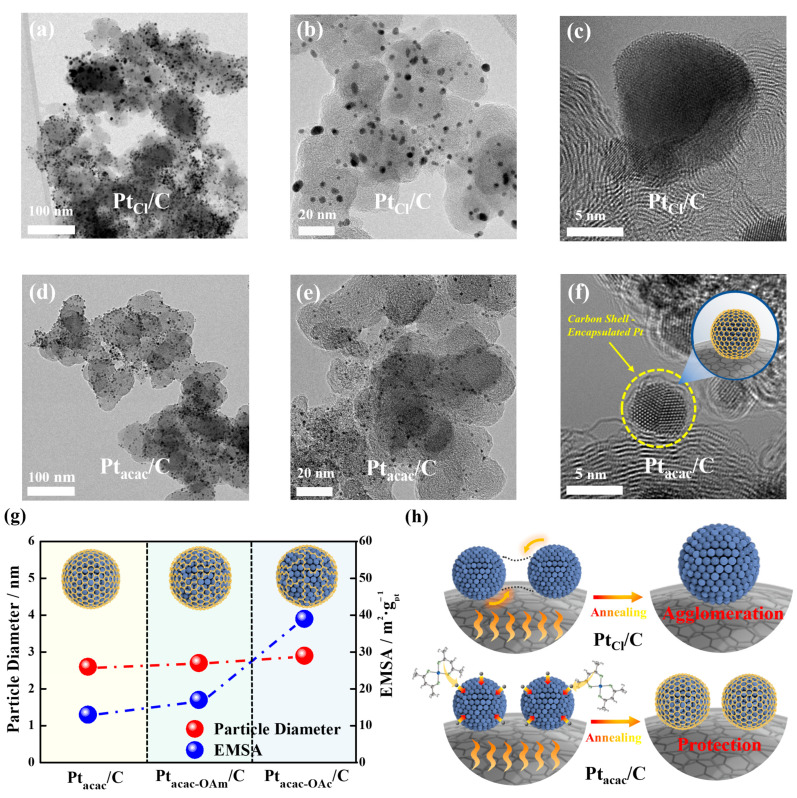
TEM images at different magnifications of (**a**–**c**) Pt_Cl_/C catalysts and (**d**–**f**) Pt_acac_/C catalysts. (**g**) Correlation between the particle diameters and exposed metal surface areas (EMSAs) according to the type of surfactants. (**h**) Schematic diagram of structural changes of Pt nanoparticles after annealing when using different Pt precursors.

As Figure 1 displays, TEM images show that the particle distribution for Pt_Cl_/C was not uniform, and particle size significantly increased, with an average size of up to 5.7 nm. Moreover, high-magnification images clearly demonstrate the absence of a carbon shell in Pt_Cl_/C (Figure 1a–c). In contrast, Pt_acac_/C maintained a highly uniform particle distribution, with a small particle size of around 2.6 nm even after annealing. Furthermore, high-resolution images reveal a distinct carbon shell with a thickness ranging from 0.5 to 1.0 nm (Figure 1d–f), and the STEM-EDS mapping result confirmed the carbon shell encapsulation (Figure S2). Unambiguously, the Pt_Cl_/C sample with larger particles exhibited a much higher EMSA than the Pt_acac_/C sample with smaller particles (Figure S3). This ultimately proves that carbon shells can be formed only when a metal precursor containing a carbon source (acetylacetonate) is used, and the presence of a carbon shell helps maintain uniform particle size due to the particle confinement effect even at high temperatures (Figures S4 and S5). 

To further validate that an organic ligand is necessary for carbon shell formation, we introduced a new approach using surfactants. In traditional nanoparticle synthesis, various surfactants are used to achieve uniform particle distribution [39,40,41,42]. However, from the perspective of the metal surface, the use of surfactants can lead to alterations in surface energy, affecting the thermodynamic equilibrium of nanocrystal formation. At this time, it is known that various metal complexes such as Pt-ligand complex Pt(OAm)_2_(acac) and Pt(OAm)_4_(OA)_2_ complex forms or Pt oleate form are formed, and this process is called ligand exchange [43,44,45,46]. 

Therefore, we attempted to analyze what changes in the surface caused by surfactants would actually bring about in electrochemical analysis and applied the concept of ligand exchange to study the carbon shell formation mechanism. First, we compared the EMSAs and ORR activities of Pt_acac-OAm_/C using only oleyamine and Pt_acac-OAc_/C using both oleyamine and oleic acid. As Figure 1g shows (Figures S6 and S7), the particle sizes of Pt_acac-OAm_/C and Pt_acac_/C after annealing were 2.6 nm and 2.7 nm, respectively, and their EMSAs were 15 m^2^∙g^−1^ and 20 m^2^∙g^−1^, showing a slight difference. Meanwhile, the particle size of Pt_acac-OAc_/C was confirmed to increase further to 2.9 nm, and the EMSA significantly increased to 39.2 m^2^∙g^−1^ (Figure 1g). And ORR activities also significantly increased in Pt_acac-OAm_/C and Pt_acac-OAc_/C (Figure S8), indicating that the carbon shell gradually became more porous when using oleylamine and oleic acid. This suggests that when both oleylamine and oleic acid were used as surfactants, more ligand exchange occurred, resulting in the formation of more Pt-surfactant complexes. Simultaneously, the number of carbon atoms originating from acetylacetonate during thermal decomposition much decreased, which was supported by a significant increase in the EMSAs when oleylamine and oleic acid were used together. Accordingly, we estimated that acetylacetonates could be displaced by the surfactant molecules due to ligand exchange, and the reduced number of carbon sources affects the porosity of the carbon shell. Based on the conclusions drawn so far, we have demonstrated that the carbon source contained in the precursor transforms into a highly crystalline carbon shell after high temperature annealing (Figure 1h).

### 3.2. Carbon Shell Formation Mechanism

In the previous section, we confirmed that the carbon source originates from the precursor by using different precursors. Now, to gain insight into the carbon shell formation mechanism, the crystal structure of the nanoparticles was closely investigated, and in-depth structural analysis was performed through XRD and XPS.

**Figure 2:**
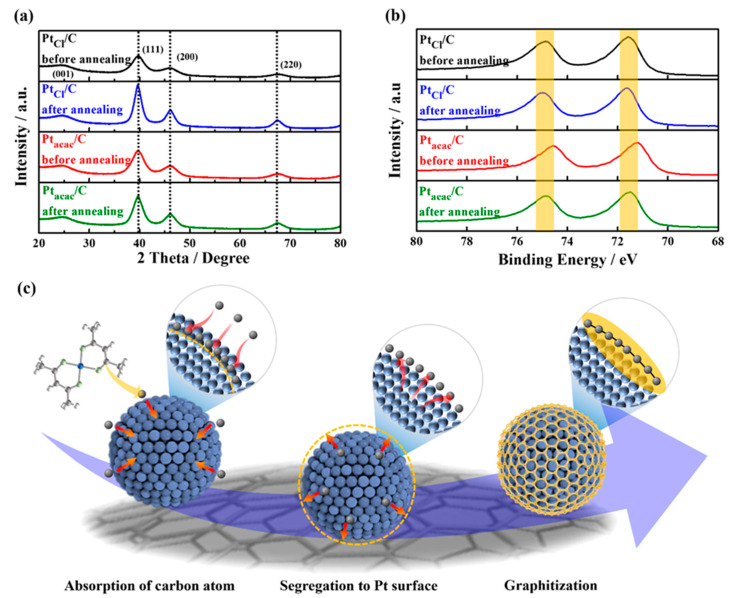
(**a**) XRD patterns and (**b**) Pt 4f core-level XPS spectra of (**b**) Pt_Cl_/C and Pt_acac_/C catalysts before and after annealing. (**c**) Scheme of carbon shell formation mechanism.

First, in the XRD patterns of the four samples (Figure 2a), from the full width at half-maximum of the Pt (220) peaks, it was confirmed that the average crystallite size of Pt_Cl_/C obviously became larger than Pt_acac_/C after annealing due to the absence of the protective effect by carbon shells, which complemented the results of the TEM analysis (Table S2). However, apart from the difference in peak sharpness, all samples exhibited similar 2 theta degrees for each XRD peak with no noticeable peak shifts compared to a commercial Pt/C catalyst. In particular, the Pt diffraction peaks, even for the Pt_acac_/C samples before and after annealing, did not shift, indicating there was no change in bulk lattice interatomic spacing. Based on XRD analysis alone, it was thought that there were no structural changes before and after carbon shell formation, except for particle size. 

However, interestingly, XPS analysis revealed that before annealing the samples, only the spectrum of the Pt_acac_/C catalyst appeared in the lower binding energy region compared to other catalysts. Subsequently, after annealing, the XPS peaks of Pt_acac_/C returned to the same position as the other samples with high binding energy. This suggests that carbon atoms entered the metal lattice near the Pt surface and increased the interatomic spacing of the sub-surface Pt atoms, resulting in tensile strain. Consequently, the d-band center upshifted, causing a right shift in binding energy in XPS [47,48,49]. On the other hand, Pt_Cl_/C showed no peak shifts or binding energy changes in both XRD and XPS, suggesting that such XPS peak shifts could be attributed to the formation of the carbon shell. 

Further detailed information can be found in the XPS fitting results, as Figure S9 and Table S1 show. As observed in the fitting results, other samples all exhibited the binding energy for Pt^0^ at 71.6 eV, whereas only the Pt_acac_/C sample before annealing had the binding energy for Pt^0^ at 71.3 eV, with both 4f_5/2_ and 4f_7/2_ peaks positioned approximately 0.3 eV lower in binding energy [50,51]. Hence, the XPS and XRD analysis results suggest that carbon atoms are more likely to be absorbed near the Pt surface rather than deep within the lattice during the thermal decomposition synthesis. Subsequently, they segregate to the outer surface during the post-annealing process at 700 °C, eventually forming a carbon shell. As a result, we can infer a series of mechanisms similar to the illustration in Figure 2c (adsorption of carbon atoms → segregation to Pt surface → graphitization). These findings shed light on the intricate process of carbon shell formation on nanoparticles.

### 3.3. Utilization of Structure-Controlled Carbon Shells

**Figure 3:**
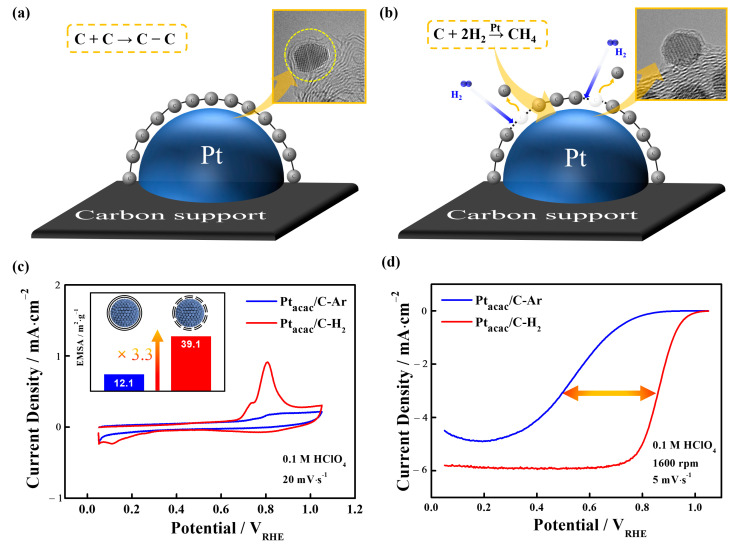
Changes in the carbon shell structure depending on the annealing gas: (**a**) Ar gas and (**b**) H_2_ gas. (**c**) CO stripping curves of Pt_acac_/C-Ar and Pt_acac_/C-H_2_. The inset shows the exposed metal surface areas (EMSAs) of the corresponding samples. (**d**) ORR polarization curves of the samples.

Up to this point, we have investigated the origin and formation mechanism of the carbon shell using various approaches and in-depth physical analyses. As with the fundamental question, the utilization of the carbon shell will also be very important. In this viewpoint, some studies have focused on the utilization perspective of the carbon shell. According to these studies, it has been determined that the carbon atoms absorbed within the lattice must undergo a segregation at the Pt surface and subsequently engage in a graphitization process forming a chemical bonding of carbon. And it typically requires high temperature annealing in the range of 500 to 1000 °C [22,23,47]. As Figure 3a shows, non-reactive gases like Ar gas during this process can lead to the formation of a dense carbon shell (see the yellow circle in the TEM image of Figure 3a) without reacting with the Pt surface. In contrast, the presence of H_2_ gas during annealing results in the creation of CH_4_ gas due to the reaction between the surface-segregated carbon atoms and H_2_ gas (Figure 3b) [52,53,54,55]. Considering the reverse mechanism of carbon shell formation, in this study, it is highly reasonable to interpret that the reaction of H_2_ gas influences the carbon shell formation.

We sought to further confirm this phenomenon through electrochemical tests. Figure 3a,b present the results of annealing conducted at 700 °C in the Ar gas atmosphere for the Pt_acac_/C-Ar sample and the H_2_ gas atmosphere for the Pt_acac_/C-H_2_ sample. First, in Figure 3c, Pt_acac_/C-Ar shows a very small EMSA of 12 m^2^∙g^−1^, suggesting the formation of a dense carbon shell (Figure S10). As demonstrated in the reference, the dense carbon shell exhibits exceptionally high selectivity for H_2_ over O_2_, resulting in a low oxygen reduction reaction (ORR) but an excellent hydrogen oxidation reaction (HOR) [24,26]. Conversely, when H_2_ gas is used, a significant increase in the EMSA is observed, indicating that H_2_ gas etches the bonding of carbons, leading to a substantial increase in Pt active sites. Therefore, the use of H_2_ gas can enhance the porosity of the carbon shell, providing a higher surface area for electrochemical reactions, particularly in ORR.

Indeed, as Figure 3d shows, when examining ORR activities, Pt_acac_/C-H_2_ with porous carbon shells due to H_2_ gas demonstrates a remarkable improvement in ORR activities, with a half-wave potential of 0.860 V compared to Pt_acac_/C-Ar, which exhibits a potential of 0.575 V. In conclusion, the use of the non-reactive gas, Ar, results in the construction of a dense carbon shell, while H_2_ gas increases carbon porosity and provides a suitable number of active sites through etching effects. 

**Figure 4:**
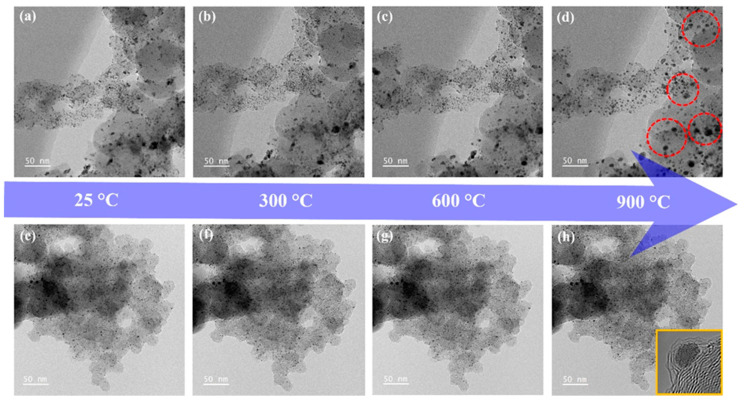
Thermal stability analysis of Pt/C and Pt_acac_/C-H_2_ catalysts using in situ TEM. Structural change in (**a**–**d**) the commercial Pt/C and (**e**–**h**) Pt_acac_/C-H_2_ depending on heating temperature.

Furthermore, when examining the TEM images of the Pt_acac_/C-H_2_, it was confirmed that the particle size was less than 5 nm, despite significant surface modification by H_2_ gas etching (Figure S11). This indicates that H_2_ gas treatment effectively secures active sites and elevates ORR activity while maintaining thermal stability in carbon shell-encapsulated catalysts. These points were further confirmed with in situ TEM analysis, which provided a clear understanding of the thermal stability of catalytic particles in the presence of a carbon shell. Firstly, as Figure 4a–d show, the commercial Pt/C began to exhibit gradual agglomeration of particles from ~400 °C when increasing the temperature from 25 to 900 °C in real-time observations. Also, as the temperature rises, particles agglomerate in several places (Figure S12).

In contrast, Pt_acac_/C-H_2_ with carbon shells were expected to provide a protective effect against agglomeration (Figure 4e,f). Indeed, it remained highly stable with no significant changes in particle size or distribution even at the high temperature of 900 °C (Figure S13). Therefore, through in situ TEM analysis, it was demonstrated that carbon shell-encapsulated catalysts are remarkably stable and can maintain strong durability through a protective effect.

**Figure 5:**
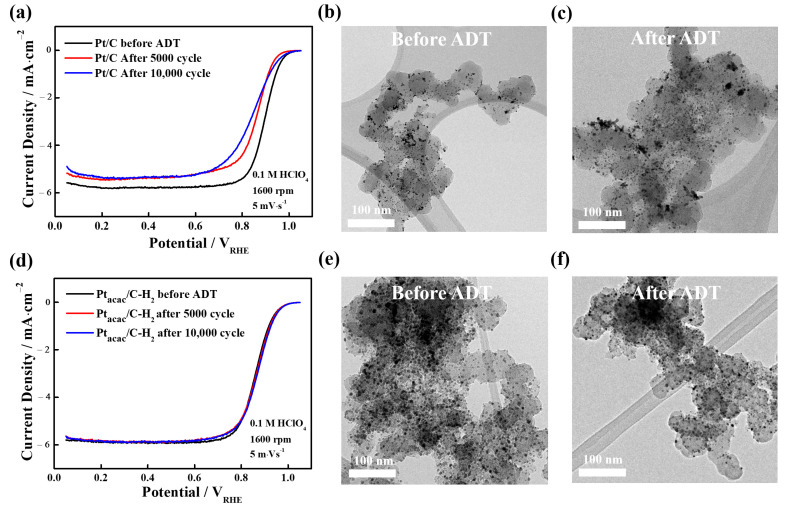
(**a**) Changes in ORR performance of the Pt/C catalysts before/after ADTs (5000 and 10,000 cycles). TEM images of the Pt/C catalysts (**b**) before and (**c**) after 10,000 cycle ADTs. (**d**) Changes in ORR performance of the Pt_acac_/C-H_2_ catalysts before/after ADTs (5000 and 10,000 cycles). TEM images of the Pt_acac_/C-H_2_ catalysts (**e**) before and (**f**) after 10,000 cycle ADTs.

Subsequently, ADTs were performed in an O_2_-saturated 0.1 M HClO_4_ electrolyte within the range of 0.6–1.1 V vs. RHE over 5000 and 10,000 cycles to confirm the electrochemical durability of Pt/C and Pt_acac_/C-H_2_. As a result, in this work, the commercial Pt/C catalyst suffered from severe particle agglomeration and dissolution of Pt, leading to a drastic performance degradation under the harsh conditions. In sharp contrast, the structure of the carbon shell-encapsulated Pt (Pt_acac_/C-H_2_) catalyst hardly changed and its ORR performance was maintained even after the ADT, demonstrating the protective effect by the carbon shell. In detail, the Pt/C catalyst had already a significant decrease in ORR activities and EMSA even after 5000 cycles, and the EMSA and ORR activity decreased further after 10,000 cycles of ADTs (Figure 5a). As Figure S14 shows, the EMSA of Pt/C was 99.7 m^2^∙g^−1^ before ADTs but was lowered to 53.7 m^2^∙g^−1^ after 10,000 cycles. In fact, TEM observation also revealed irregularities in particle distribution and increased particle size after ADTs, as Figure 5b,c show. On the other hand, for the Pt_acac_/C-H_2_, there was no decrease in ECSA and ORR activities after the ADTs, as Figure 5d shows (Figures S15 and S16). And TEM observations also confirmed that the particle distribution and size remained relatively consistent (Figure 5e,f). Consequently, it can be concluded that when a carbon shell with suitable porosity is employed, not only is ORR activity improved but durability is also significantly enhanced [18,19,56].

## 4. Conclusions

In summary, we explored the origin of carbon shells by utilizing various precursors to determine whether carbon shells originate from precursors containing a carbon source. Through TEM analysis and the introduction of a novel approach known as ligand exchange, this was convincingly demonstrated. Furthermore, through in-depth physical analyses such as XRD and XPS, it was suggested that carbon atoms are absorbed near the Pt surface rather than deep within the lattice during the thermal decomposition synthesis, and segregate to the outer surface during post-annealing, eventually forming a carbon shell. Moreover, it was confirmed that the porosity (or density) of the carbon shell can change depending on the type of annealing gas. Lastly, the present study confirms the thermal stability and electrochemical durability of carbon shells through in situ TEM and ADTs, ultimately demonstrating that carbon shell encapsulation is highly effective in addressing the long-term stability issues for fuel cells. Accordingly, it is believed that valuable insights can be provided for the development of structural control strategies for electrocatalysts. Additionally, it is anticipated that the utilization of thermal stability may not only benefit electrocatalysts but also extend to thermochemical catalysts for various chemical reations.

## Data Availability

No data were used for the research described in the article.

## Supplementary Materials

The following supporting information can be downloaded at: https://www.mdpi.com/article/10.3390/nano13212862/s1, Figure S1: TEM images of Pt/C catalysts synthesized using different Pt precursors: (a) H_2_PtCl_6_·xH_2_O, (b) PtCl_4_, and (c) PtCl_2_. TGA curves of (d) Pt_acac_/C-Ar and (e) Pt_Cl_/C-Ar catalysts; Figure S2: STEM-EDS mapping images of the Ptacac/C sample. Green and red dots in the STEM-EDS images indicate carbon and Pt atoms, respectively; Figure S3: CO stripping curves of Ptacac/C and Pt_Cl_/C; Figure S4: TEM images at different magnifications and particle size distribution of PtCl/C catalysts (a,b) before and (c,d) after annealing at 700 °C. The particle size distribution and average particle size in the insets of Figure S4b,d were obtained by examining 30 particles in the corresponding TEM images, and the error range for the average particle size was ±0.1 and ±0.5 nm, respectively; Figure S5: TEM images at different magnifications and particle size distribution of Ptacac/C catalysts (a,b) before and (c,d) after annealing at 700 °C. The particle size distribution and average particle size in the insets of Figure S5b,d were obtained by examining 30 particles in the corresponding TEM images, and the error range for the average particle size was ±0.1 nm, respectively; Figure S6: TEM images at different magnifications and particle size distribution of Ptacac-OAm/C catalysts (a,b) before and (c,d) after annealing at 700 °C. The particle size distribution and average particle size in the insets of Figure S6b,d were obtained by examining 30 particles in the corresponding TEM images, and the error range for the average particle size was ±0.1 nm, respectively; Figure S7: TEM images at different magnifications and particle size distribution of Ptacac-OAc/C catalysts (a,b) before and (c,d) after annealing at 700 °C. The particle size distribution and average particle size in the insets of Figure S7b,d were obtained by examining 30 particles in the corresponding TEM images, and the error range for the average particle size was ±0.1 nm, respectively; Figure S8: Electrochemical properties of the Pt_acac_/C, Pt_acac-OAm_/C, and Pt_acac-OAc_/C catalysts: (a) CVs, (b) CO stripping curves, and (c) ORR polarization curves; Figure S9: Pt4f XPS spectra of (a,b) Pt_Cl_/C and (c,d) Pt_acac_/C before/after annealing; Figure S10: CVs of Pt_acac_/C-Ar and Pt_acac_/C-H_2_ catalysts; Figure S11: TEM images at different magnifications and particle size distribution of Pt_acac_/C-H_2_. The particle size distribution and average particle size in the insets of the right figure were obtained by examining 30 particles in the corresponding TEM image, and the error range for the average particle size was ±0.1 nm; Figure S12: In situ TEM images of commercial Pt/C (25 °C~900 °C); Figure S13: In situ TEM images of Pt_acac_/C-H_2_ (25 °C~900 °C); Figure S14: Electrochemical properties of the Pt/C catalysts before and after ADTs: (a) CVs, (b) CO stripping curves, and (c) EMSAs; Figure S15: Electrochemical properties of the Pt_acac_/C-H_2_ catalysts before and after ADTs: (a) CVs, (b) CO stripping curves; Figure S16: Changes in mass activity of the Pt/C and Pt_acac_/C-H_2_ catalysts before and after ADTs; Table S1: XPS data of Pt_Cl_/C and Pt_acac_/C catalysts before/after annealing; Table S2: Particle size and crystalline size of Pt_Cl_/C and Pt_acac_/C catalysts before/after annealing through to TEM and XRD.
