# Supplementary material for: Origin and Formation Mechanism of Carbon Shell-Encapsulated Metal Nanoparticles for Powerful Fuel Cell Durability
Source: Nanomaterials (Basel). 2023 Oct 29;13(21):2862. doi: 10.3390/nano13212862 (PMC10648549; doi:10.3390/nano13212862)
Supplement: Supplementary file 1 [file nanomaterials-13-02862-s001.zip › nanomaterials-2668477-supplementary.pdf]

Supplementary Information

Origin and Formation Mechanism of Carbon Shell-Encapsulated Metal Nanoparticles for Powerful Fuel

Hyeonwoo Choi, Yoonseong Choi, Jiho Min, Keonwoo Ko, Yunjin Kim, Sourabh S. Chougule, Davletbaev Khikmatulla and Namgee Jung *

Graduate School of Energy Science and Technology (GEST), Chungnam National University, 99 Daehak-ro, Yuseong-gu, Daejeon 34134, Republic of Korea; snow7780@o.cnu.ac.kr (H.C.); gubongman@o.cnu.ac.kr (Y.C.); mjh9780@o.cnu.ac.kr (J.M.); kkw00000@o.cnu.ac.kr (K.K.); yunjinkim1994@o.cnu.ac.kr (Y.K.); schougule@o.cnu.ac.kr (S.S.C.); haki030899@o.cnu.ac.kr (D.K.)

* Correspondence: njung@cnu.ac.kr

Experimental

1. Pt nanoparticles synthesis using $H_2PtCl_6{\cdot}xH_2O$ and $PtCl_4$ precursor

To synthesize the catalyst using $H_2PtCl_6{\cdot}xH_2O$ and $PtCl_4$ precursors, carbon black of 0.1g (Vulcan XC72, Cabot) were well dispersed in 1-octadecene of 140 mL (90 %, Sigma-Aldrich) by sonication for 20 min, and 0.142 g of $H_2PtCl_6{\cdot}xH_2O$ (37-40 %, Sigma-Aldrich) or 0.0432 g of $PtCl_4$ (96 %, Sigma-Aldrich) were dispersed in 20 mL of 1-octadecene by sonication for 20 min. After the two solutions were mixed and then stirred in Ar atmosphere at 120 °C for 1 h to remove H_2O impurities, the solution temperature was increased up to 300 °C and kept for 2 h for the thermal decomposition of the Pt precursor. After completing the reaction, the solution was cooled down to 80 °C and then filtered and washed by copious hexane (95.0%, Samchun Pure Chemical) and ethanol (95.0%, Samchun Pure Chemical). The as-prepared catalyst was dried in an oven at 60 °C and then annealed at 700 °C for 1 h in Ar atmosphere to form carbon shell layers on Pt nanoparticles.

2. Calculation of exposed metal surface areas (EMSAs)

The EMSAs of the prepared catalysts were calculated from CO stripping curves using the following equation.

$$EMSA(m^2\ g_{pt}^{-1}) = \frac{Total\ charge(Q)\ for\ CO\ oxidation}{Q_{CO}(420\ \mu C\ cm^{-2})\ \times\ Pt\ loading(g_{Pt})}$$

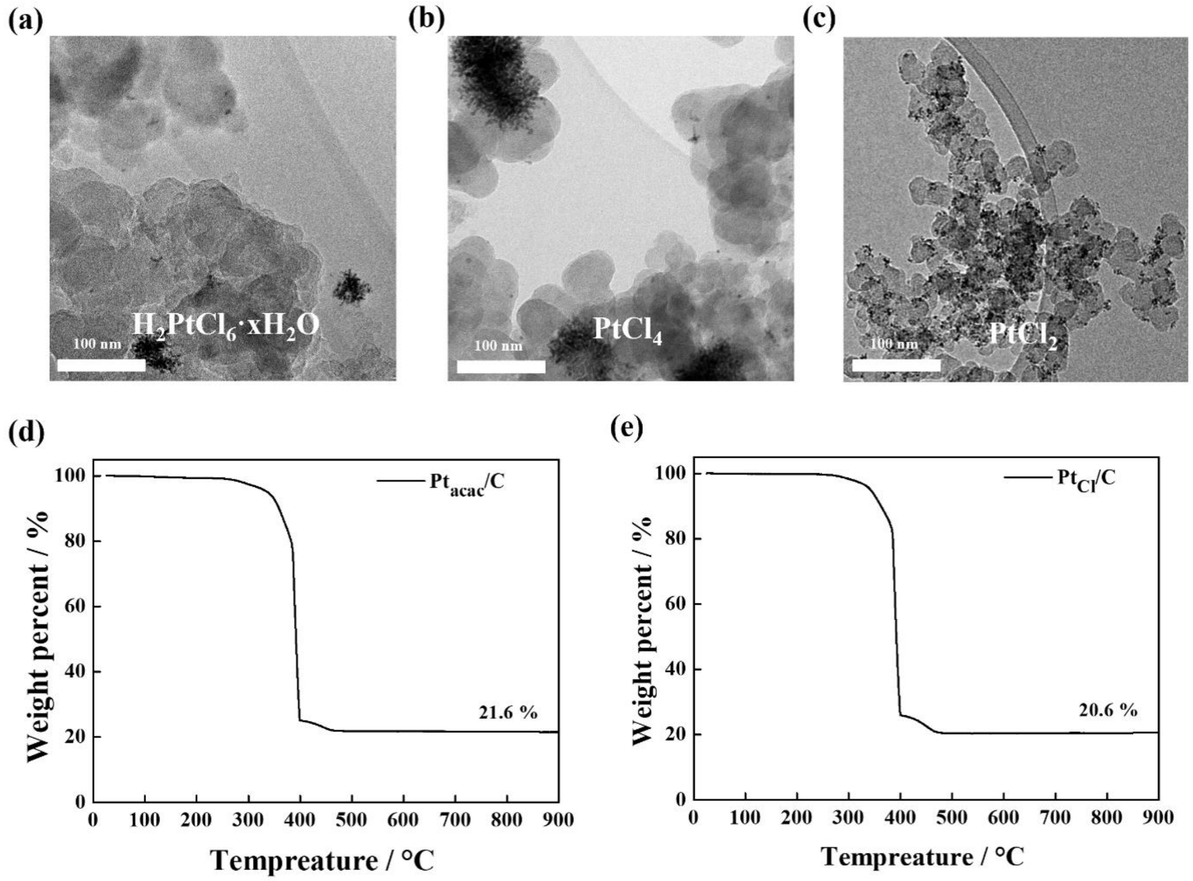

Figure S1. TEM images of Pt/C catalysts synthesized using different Pt precursors: (a) $H_2PtCl_6·xH_2O$, (b) $PtCl_4$, and (c) $PtCl_2$. TGA curves of (d) Pt_{acac}/C and (e) Pt_{Cl}/C catalysts.

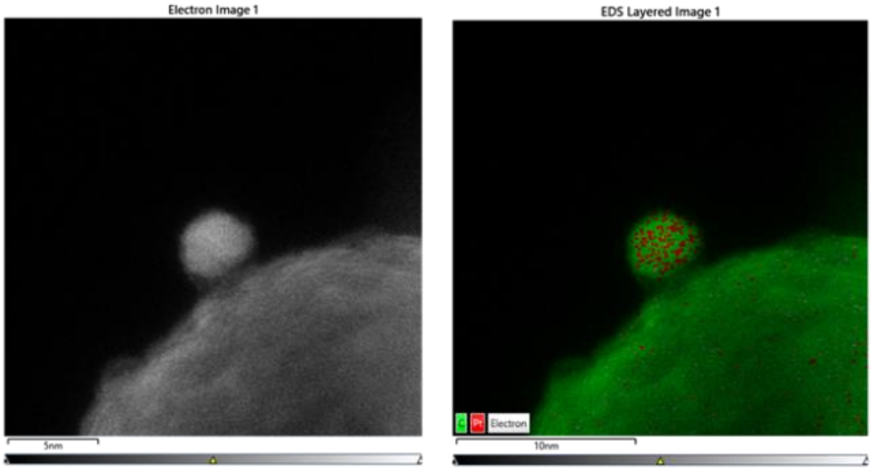

Figure S2. STEM-EDS mapping images of the Pt_{acac}/C sample. Green and red dots in the STEM-EDS images indicate carbon and Pt atoms, respectively.

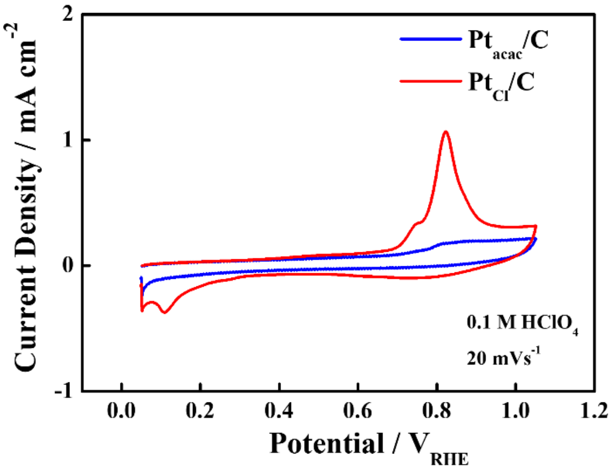

Figure S3. CO stripping curves of Pt_{acac}/C and Pt_{Cl}/C.

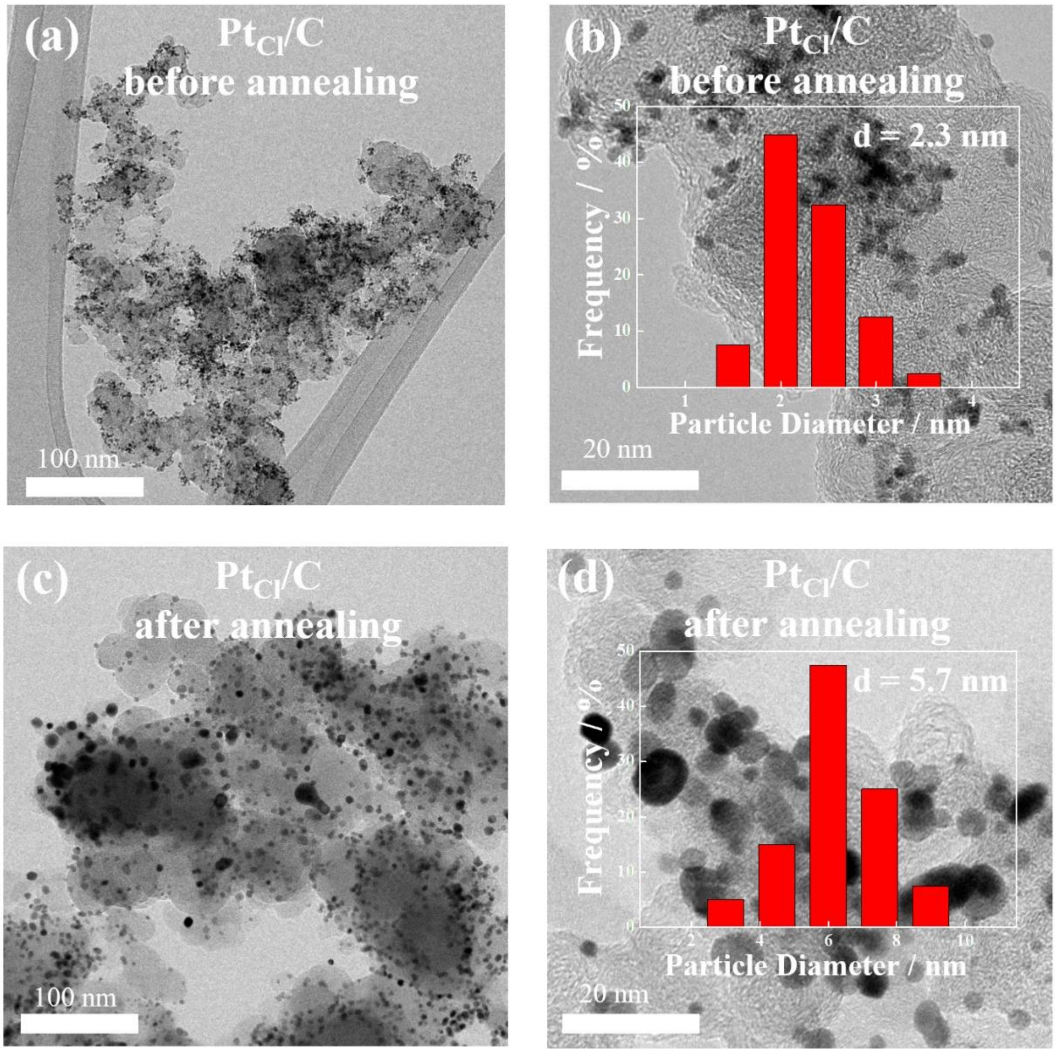

Figure S4. TEM images at different magnifications and particle size distribution of Pt_{Cl}/C catalysts (a,b) before and (c,d) after annealing at 700 °C. The particle size distribution and average particle size in the insets of Figure S4b and S4d were obtained by examining 30 particles in the corresponding TEM images, and the error range for the average particle size was ±0.1 and ±0.5 nm, respectively.

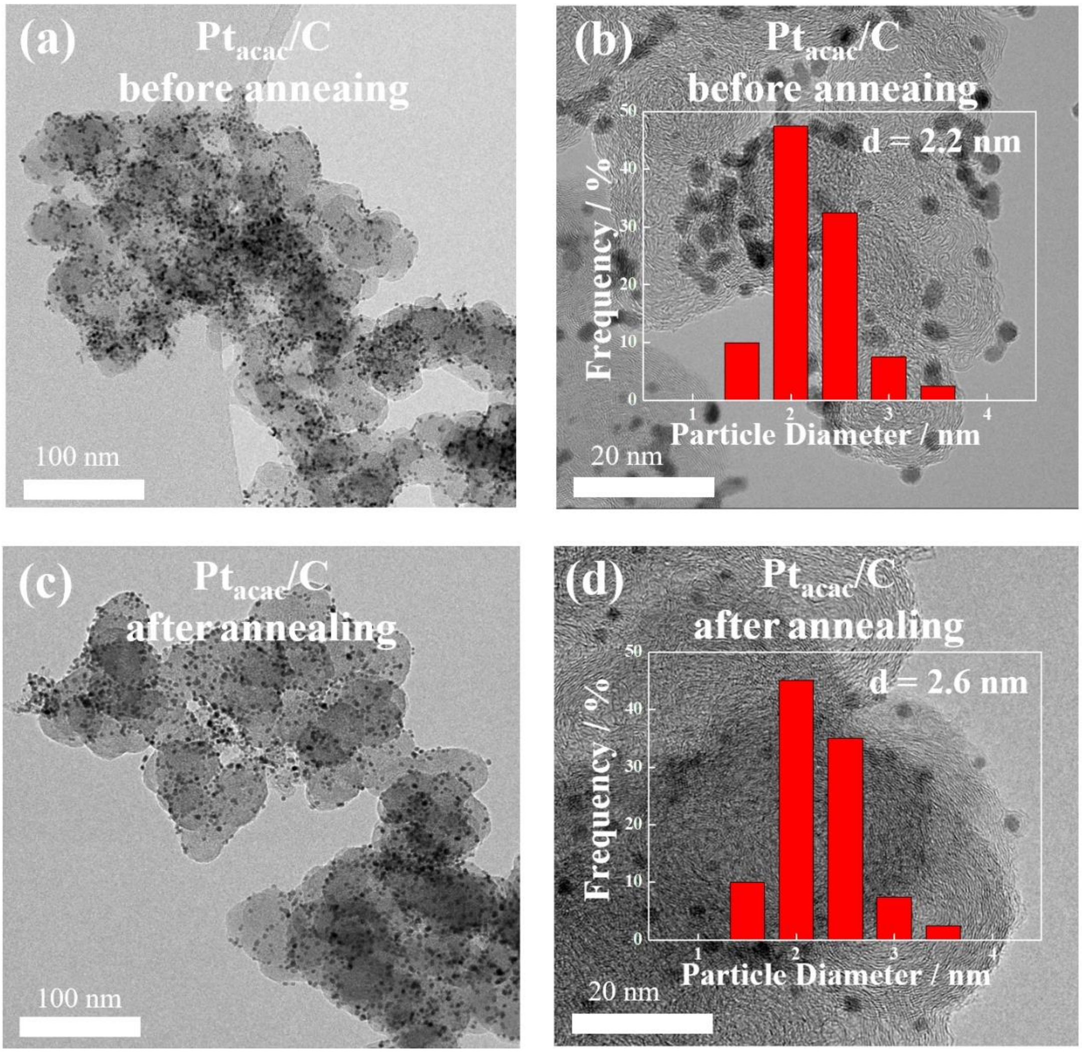

Figure S5. TEM images at different magnifications and particle size distribution of Pt$_{acac}$/C catalysts (a,b) before and (c,d) after annealing at 700 °C. The particle size distribution and average particle size in the insets of Figure S5b and S5d were obtained by examining 30 particles in the corresponding TEM images, and the error range for the average particle size was ±0.1 nm, respectively.

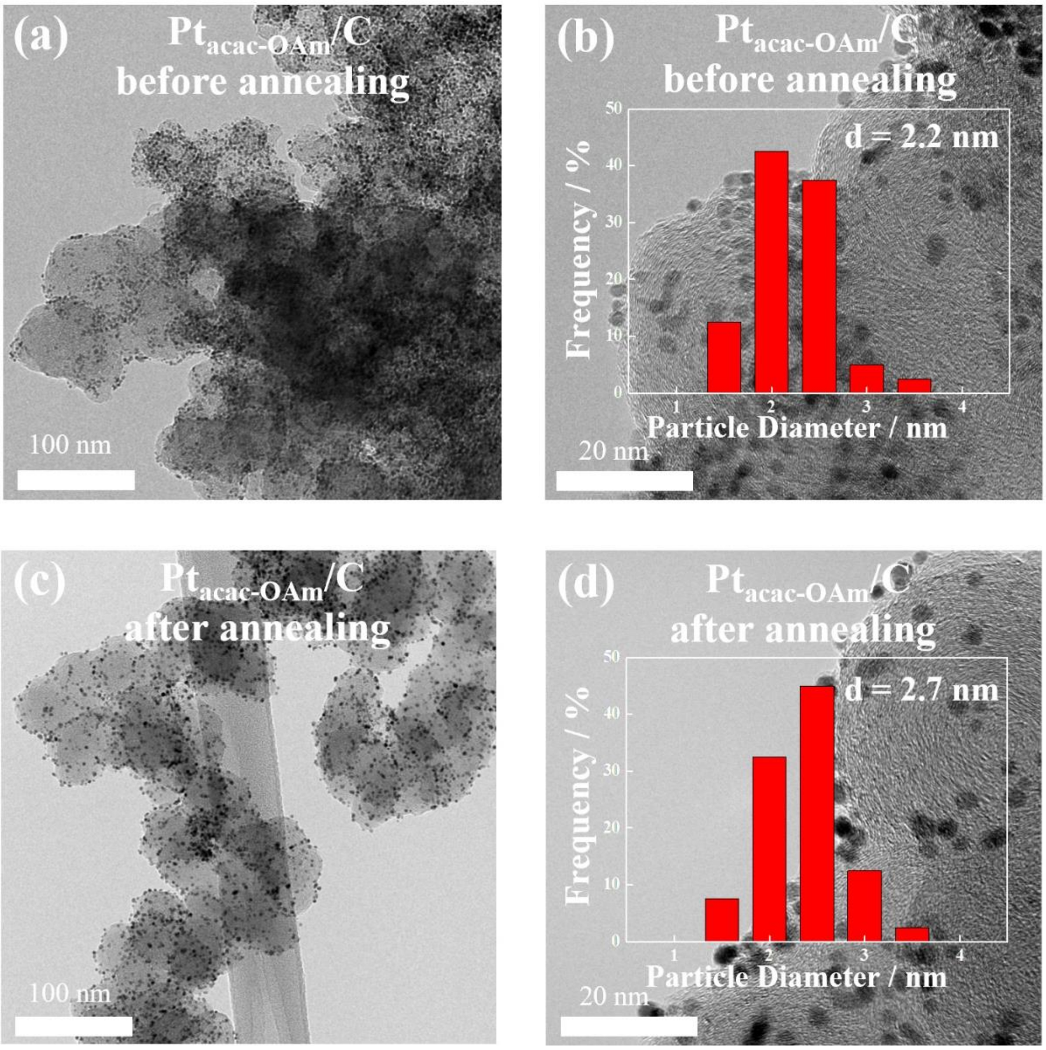

Figure S6. TEM images at different magnifications and particle size distribution of $Pt_{acac-OAm}/C$ catalysts (a,b) before and (c,d) after annealing at 700 °C. The particle size distribution and average particle size in the insets of Figure S6b and S6d were obtained by examining 30 particles in the corresponding TEM images, and the error range for the average particle size was ±0.1 nm, respectively.

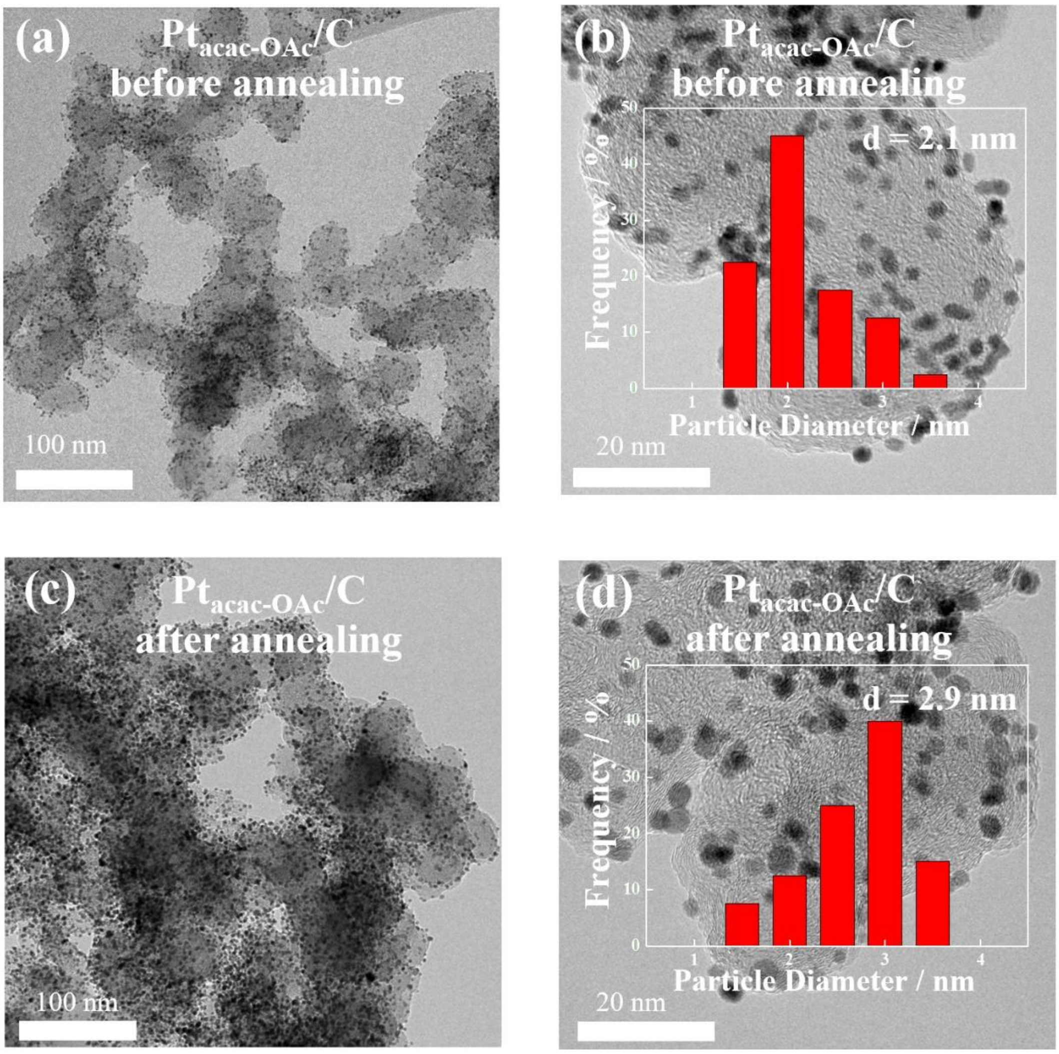

Figure S7. TEM images at different magnifications and particle size distribution of Pt$_{acac\text{-}OAc}$/C catalysts (a,b) before and (c,d) after annealing at 700 °C. The particle size distribution and average particle size in the insets of Figure S7b and S7d were obtained by examining 30 particles in the corresponding TEM images, and the error range for the average particle size was ±0.1 nm, respectively.

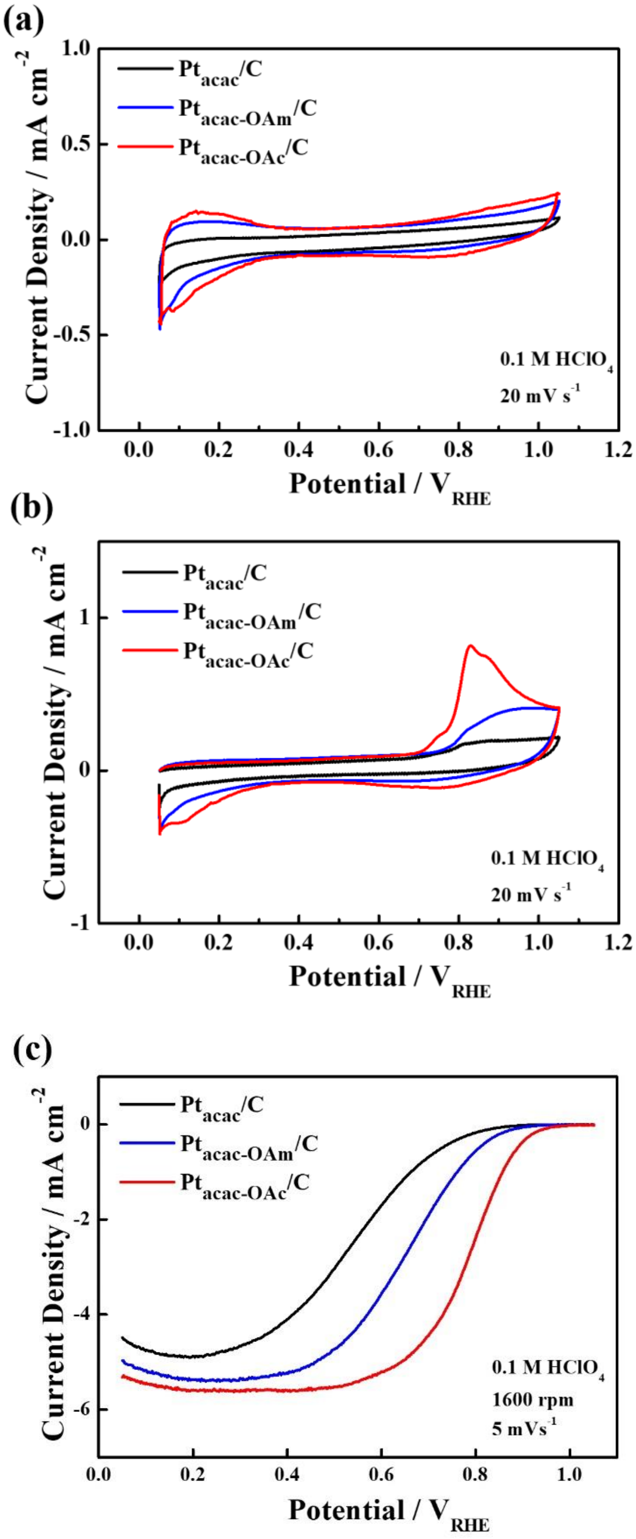

Figure S8. Electrochemical properties of the Pt_{acac}/C, $Pt_{acac-OAm}$/C, and $Pt_{acac-OAc}$/C catalysts: (a) CVs, (b) CO stripping curves, and (c) ORR polarization curves.

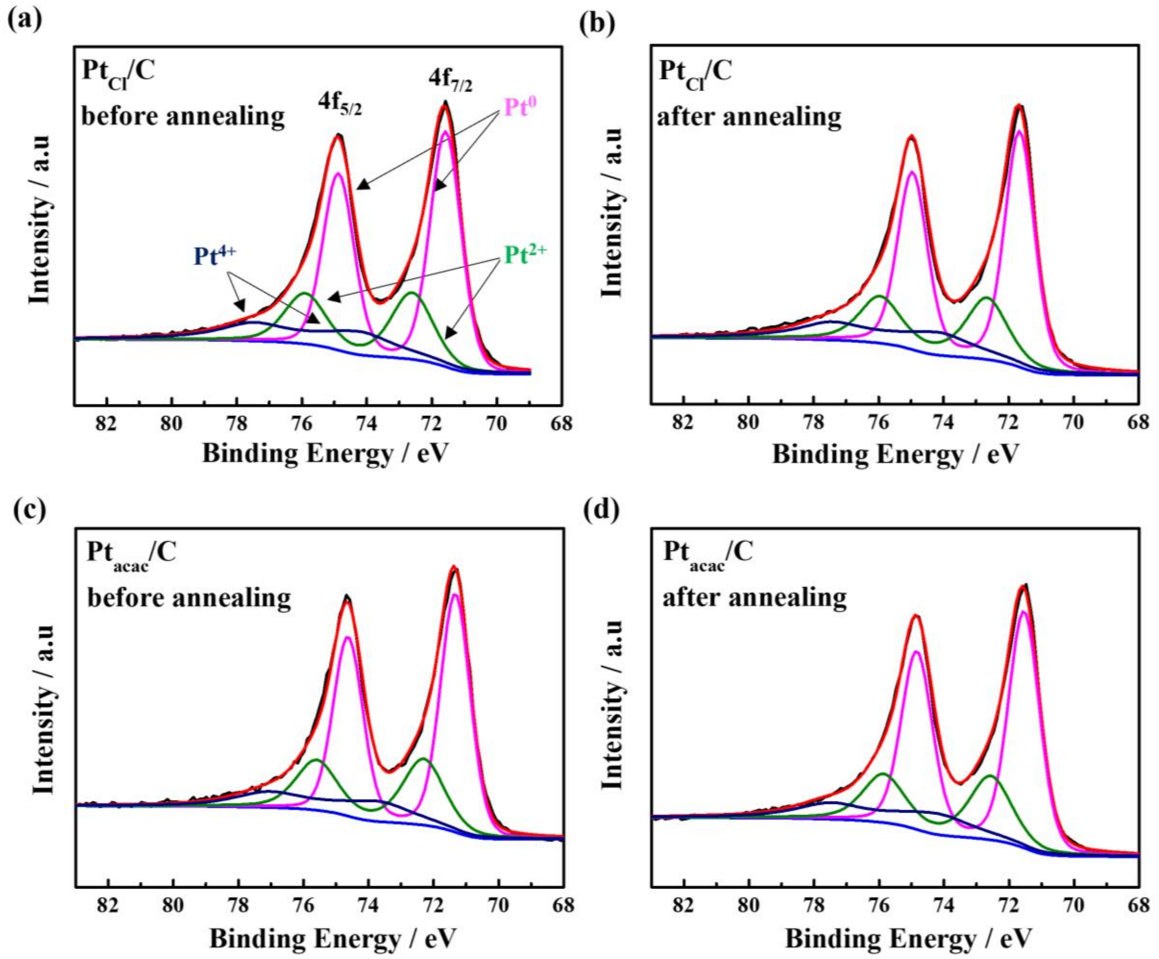

Figure S9. Pt4f XPS spectra of (a,b) Pt_{Cl}/C and (c,d) Pt_{acac}/C before/after annealing.

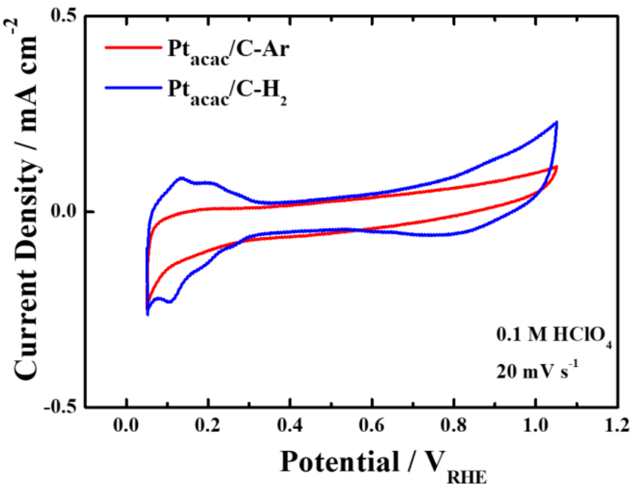

Figure S10. CVs of Pt_{acac}/C-Ar and Pt_{acac}/C-H_2 catalysts.

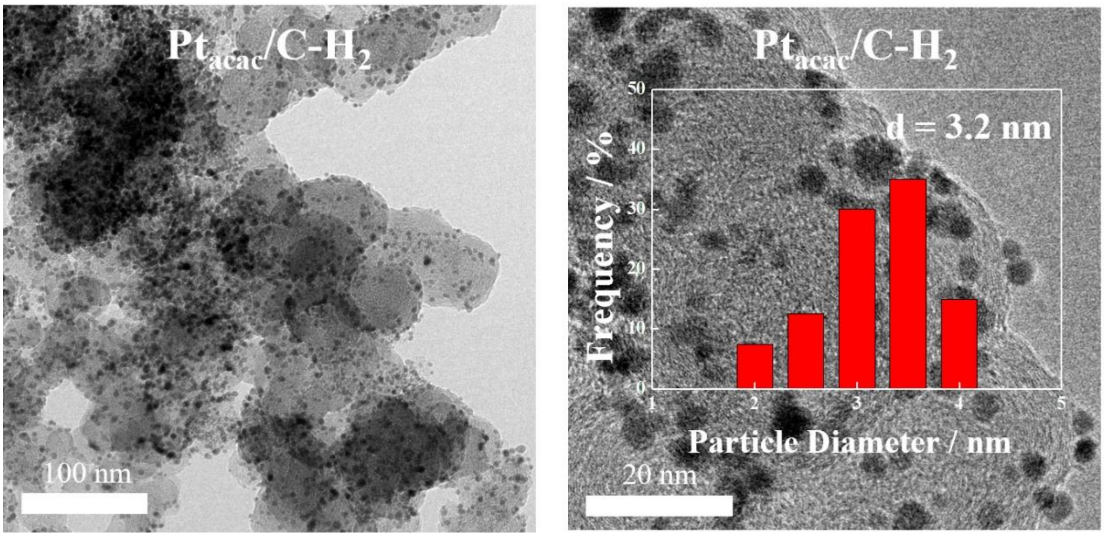

Figure S11. TEM images at different magnifications and particle size distribution of $Pt_{acac}/C\text{-}H_2$. The particle size distribution and average particle size in the insets of the right figure were obtained by examining 30 particles in the corresponding TEM image, and the error range for the average particle size was ±0.1 nm.

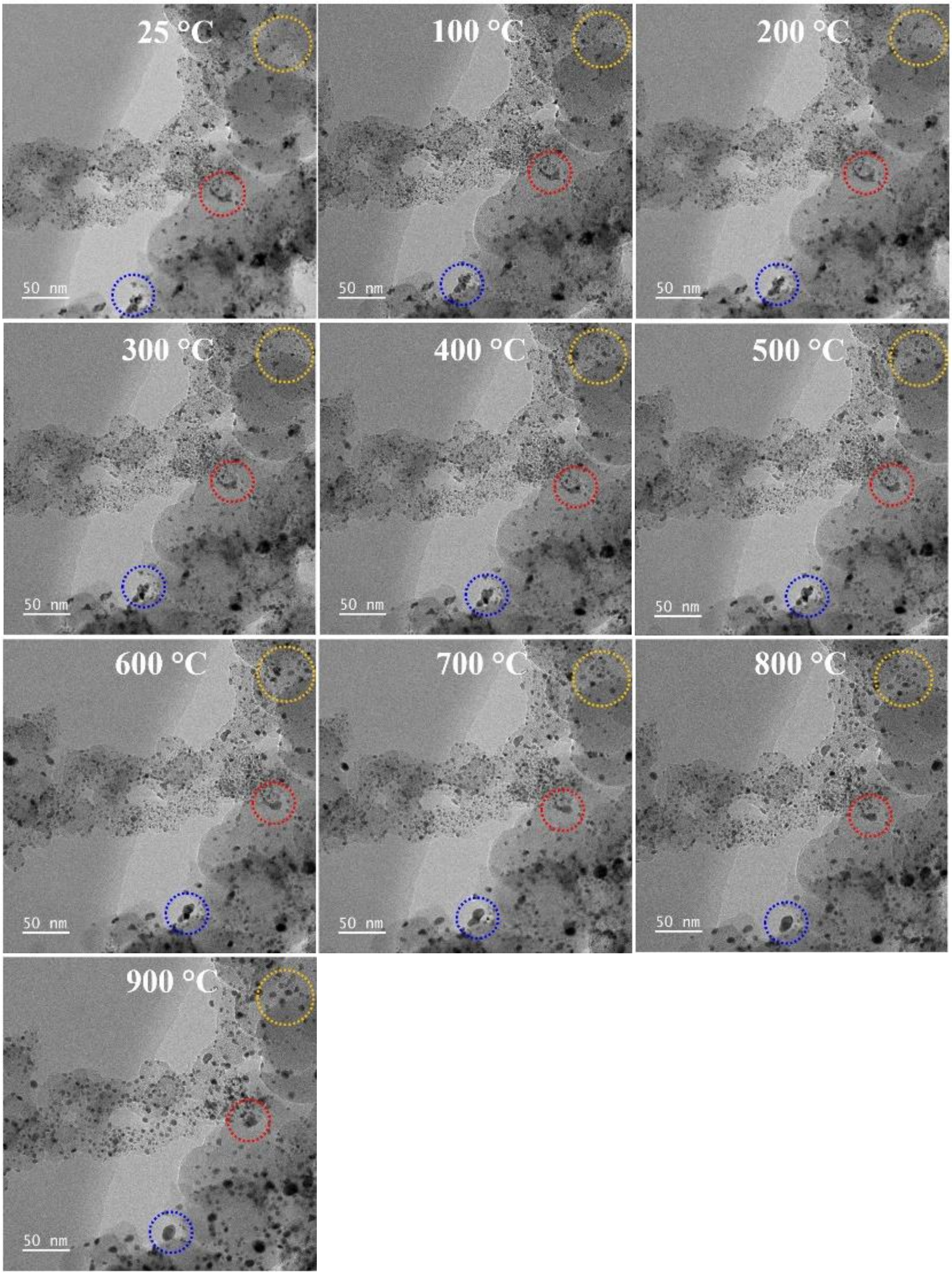

Figure S12. In situ TEM images of commercial Pt/C (25 °C ~ 900 °C).

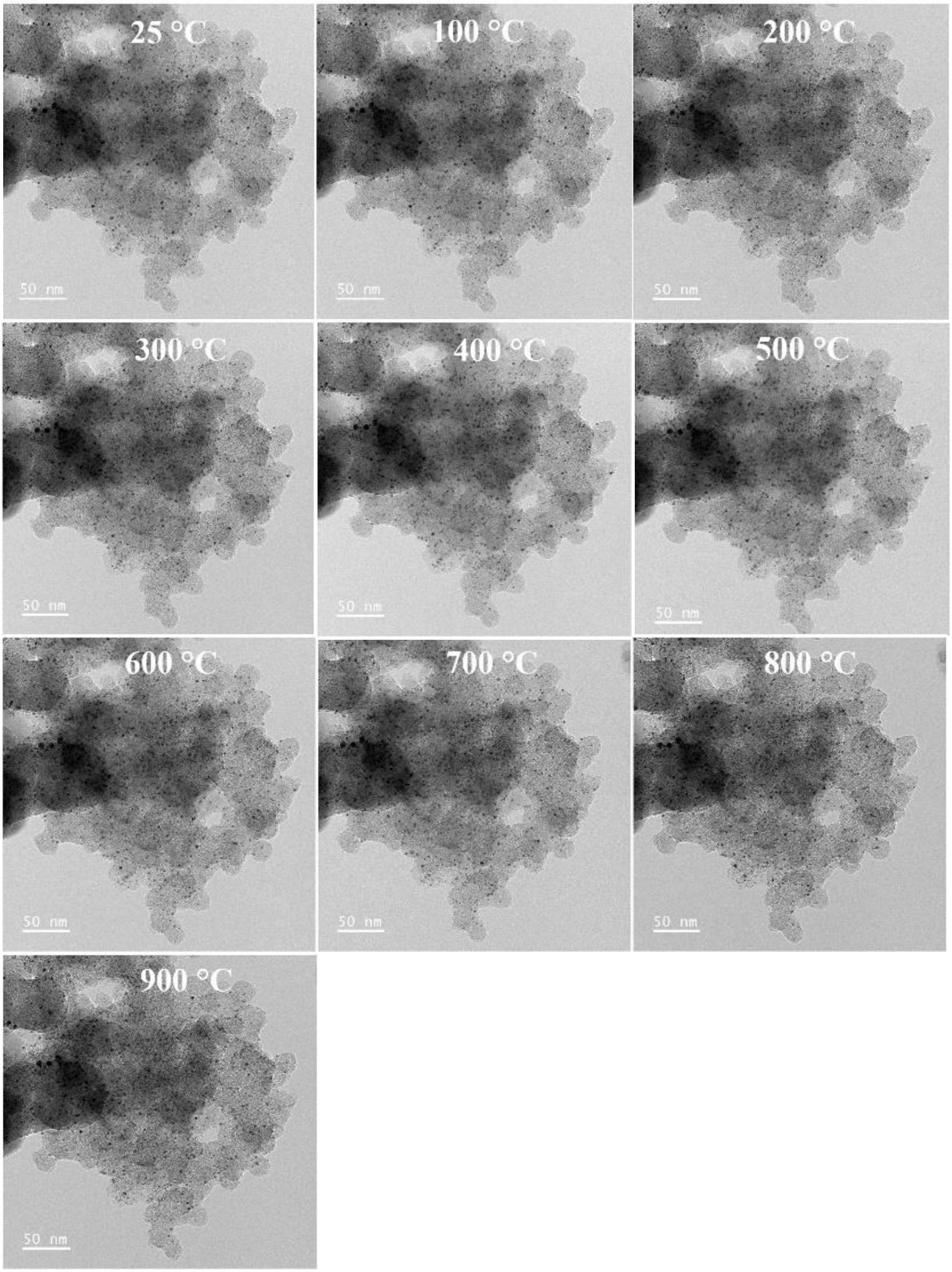

Figure S13. In situ TEM images of Pt_{acac}/C-H_2 (25 °C ~ 900 °C).

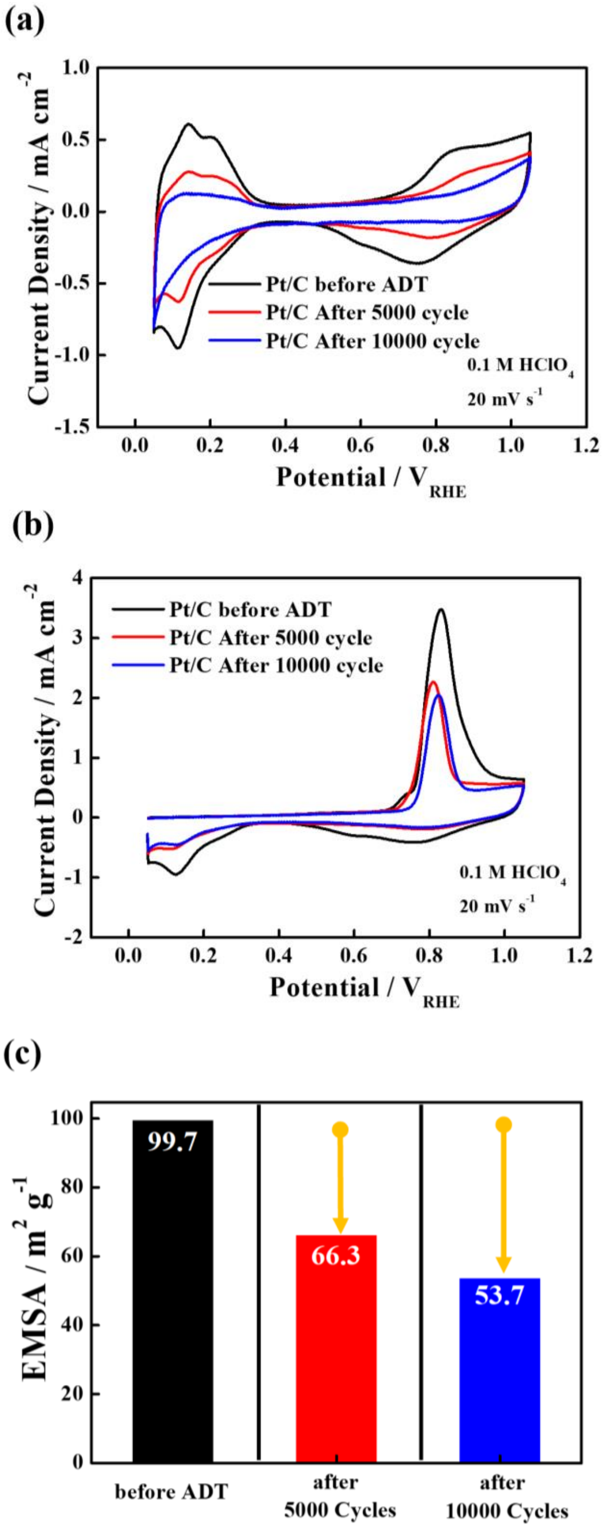

Figure S14. Electrochemical properties of the Pt/C catalysts before and after ADTs: (a) CVs, (b) CO stripping curves, and (c) EMSAs.

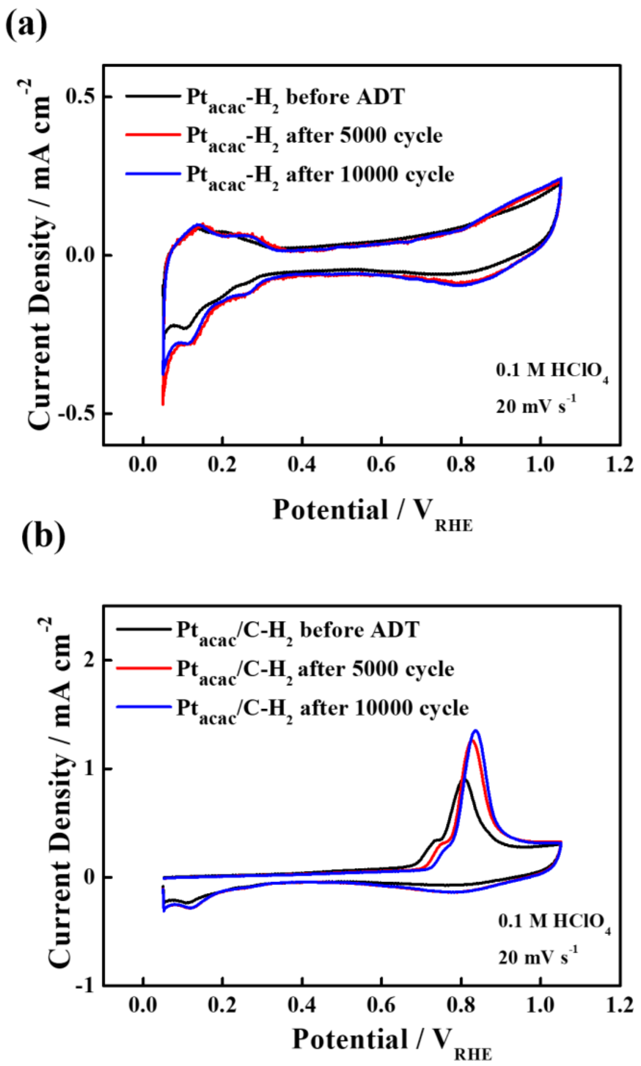

Figure S15. Electrochemical properties of the Pt$_{acac}$/C-H$_2$ catalysts before and after ADTs: (a) CVs and (b) CO stripping curves.

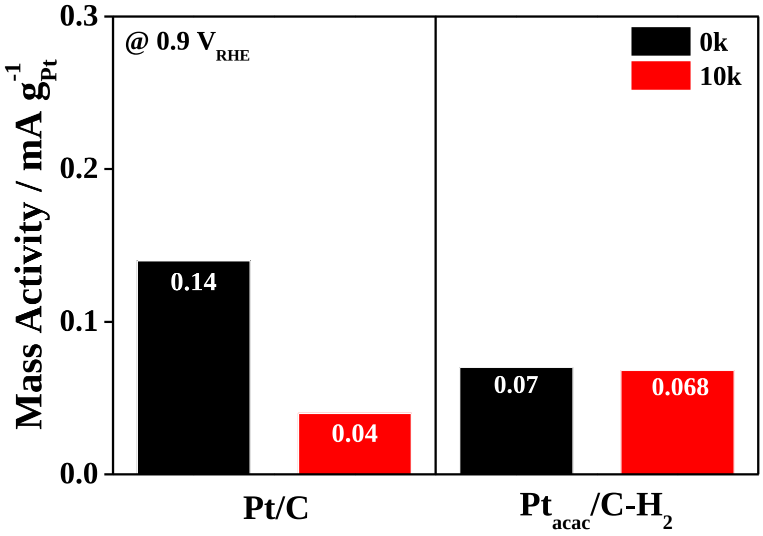

Figure S16. Changes in mass activity of the Pt/C and and Pt_{acac}/C-H_2 catalysts before and after ADTs.

Table S1. XPS data of Pt_{Cl}/C and Pt_{acac}/C catalysts before/after annealing.

Catalysts	Peak	Binding energy (eV)	Oxdiation state	Relative ratio (%)
Pt_{Cl}/C before annealing	Pt 4f	71.57	Pt^0	59.6
		72.6	Pt^{2+}	24.1
		74.2	Pt^{4+}	16.3
Pt_{Cl}/C after annealing	Pt 4f	71.64	Pt^0	60.1
		72.66	Pt^{2+}	24.1
		74.15	Pt^{4+}	15.8
Pt_{acac}/C before annealing	Pt 4f	71.33	Pt^0	60.5
		72.3	Pt^{2+}	25.4
		73.75	Pt^{4+}	14.1
Pt_{acac}/C after annealing	Pt 4f	71.56	Pt^0	61.4
		72.55	Pt^{2+}	24.5
		74.2	Pt^{4+}	14.1

Table S2. Particle size and crystalline size of Pt_{Cl}/C and Pt_{acac}/C catalysts before/after annealing through to TEM and XRD.

catalysts	TEM (Particle size)	XRD (Crystalline size)
Pt_{Cl}/C before annealing	2.3 nm	2.1 nm
Pt_{Cl}/C after annealing	5.7 nm	3.5 nm
Pt_{acac}/C before annealing	2.2 nm	2.2 nm
Pt_{acac}/C after annealing	2.6 nm	2.5 nm
